# An Evaluation of the Effectiveness of Various Luting Cements on the Retention of Implant-Supported Metal Crowns

**DOI:** 10.7759/cureus.41691

**Published:** 2023-07-11

**Authors:** Surbhi Mehta, Anubhav Kesari, Mohit Tomar, Urvashi Sharma, Preeti Sagar, Pooja Nakum, Kumuda Rao

**Affiliations:** 1 Prosthodontics and Crown & Bridge, Inderprastha Dental College & Hospital, Ghaziabad, IND; 2 Prosthodontics and Crown & Bridge, Subharti Dental College, Meerut, IND; 3 Prosthodontics and Crown & Bridge, Smile Carve Dental Clinic, Jaipur, IND; 4 Prosthodontics and Crown & Bridge, Homi Bhabha Cancer Hospital & Research Center-Muzaffarpur (A Unit of Tata Memorial Hospital, Mumbai), Muzaffarpur, IND; 5 Oral Surgery, Goenka Research Institute of Dental Science, Gandhinagar, IND; 6 Oral Medicine and Radiology, AB Shetty Memorial Institute of Dental Sciences - Nitte (Deemed to be University), Mangalore, IND

**Keywords:** resin cement, retention test, luting, implant, cement

## Abstract

Background and objective

Cement-retained prostheses have replaced screw-retained prostheses as the preferred restoration in recent years in order to overcome the latter's limitations. In this study, four different luting cements were compared to evaluate their efficacy on the retention of cement-based metal crowns to implant abutments.

Materials and methods

In the right and left first molar regions, four implant analogs (Internal Hex, Adin Dental Implant Systems Ltd., Tel-Aviv, Israel) were screwed into epoxy resin casts (Araldite CY 230-1 IN, India) that were positioned perpendicular to the cast's plane. Four metal copings were created and cemented. Group A: polycarboxylate cement (DUR) (DurelonTM, 3M Espe, St. Paul, MN); Group B: PANAVIA™ F 2.0 dual-cure resin cement (Kuraray America, Inc., New York, NY); Group C: resin-modified glass ionomer (3M™ RelyX™ Luting, 3M Espe); and Group D: non-eugenol temporary resin cement (Kerr-Temp, KaVo Kerr, Brea, CA) were used to cement crowns. To check the retention capacity, samples were put through a pull-out test on an Instron universal testing machine (TSI‑Tecsol, Bengaluru, India) with a crosshead speed of 0.5 mm/min. Each coping's de-cementing load was noted, and average values for every sample were computed and statistically analyzed.

Results

The findings demonstrated that non-eugenol temporary resin implant cement has the lowest retention value at 138.256 N, followed by resin-modified glass ionomer cement at 342.063 N, polycarboxylate luting cement at 531.362 N, and resin cement at 674.065 N. The average difference in retentive strength across all four groups was statistically very significant (p=0.001).

Conclusion

Based on our findings, non-eugenol temporary resin implant cement enables simple retrievability of the prosthesis in the event of a future failure and is appropriate for implant restorations with cement retention. Also, cements made of polycarboxylate and resin have the highest retention values.

## Introduction

The advent of dental implants has improved the treatment options in prosthetic dentistry [1]. The majority of implant-supported prostheses are fixed restorations, and they can replace one or more missing teeth. Prosthetic screws or cement retention are the two ways to keep the suprastructure attachment to the implant abutment constituent. When restoring a full-arch implant, the screw-retained prosthesis is typically the retention method of choice [2]. Tighter margins are produced by crowns supported by implants with screws. However, there is more stress concentrated around the implants as a result of the screw-retained superstructures' lack of passive fit. Due to the direction and magnitude of oral forces as well as the strength restrictions of the components, implant screw joints are vulnerable to screw loosening or fracture, which is one of the drawbacks of screw-retained prostheses. Managing screw-related complications can be challenging, particularly if the prosthesis has been permanently anchored [3].

Cement-retained prostheses have replaced screw-retained prostheses as the preferred method of restoration for the care of implant patients in order to overcome the latter's limitations [3]. In comparison to screw-retained restorations, cemented restorations are a more popular alternative. The benefits they provide include the elimination of prosthesis screw loosening, decreased chair-side time, and streamlined clinical and treatment planning processes [1,2]. Compared to screw-retained prostheses, cemented prostheses exhibit better occlusion and aesthetics, as well as easier laboratory procedures and loading characteristics. However, cemented prostheses' primary drawback is their irretrievability [2,3].

Temporary luting cements are the most commonly used cements for the retention of implant prostheses. The selection of cement is crucial for achieving an appropriate level of retention of the implant prosthesis while allowing for removal, thereby increasing the longevity of implant prostheses. The most frequently used cement for implant prosthesis retention is temporary luting cement. The ideal cement type must be sufficient enough to keep the crown in place for an indefinite period, but weak enough to enable the dentist to remove it if required [2]. According to reports, external forces, cement space or internal relief, type of luting agent, and cement material selection can all affect how retentive the final restorations are [2]. The retention strength of the cement used is inversely correlated with the degree of retrievability of the implant-supported cement-retained prosthesis. The practitioner can determine whether the degree of retention is sufficient to prevent debonding while facilitating retrieval, if necessary, by using the retention strength data for specific cements [4].

The luting agents that are currently available and used clinically to attach crowns to implant abutments include resin-based urethane-based cement (temporary cement), resin-modified glass ionomer, resin composite cements (permanent cements), and others [4]. A dual-cured resin composite made of dimethacrylate resin, ytterbium trifluoride fillers, barium glass, and HEMA make up the bonding cement. They have superior flexural, compressive, and tensile strengths, less solubility or water absorption, and greater bonding strength than resin-modified glass ionomer cements [4]. Zinc oxide eugenol (ZOE) cement (TempBond^TM^) is known to have high solubility when in direct contact with water and to require enough time for a full setting reaction, while that is not the case with non-eugenol zinc oxide resin cement [2].

In this study, polycarboxylate, resin-modified glass ionomer cement, dual cure resin cement, and non-eugenol temporary resin cement were compared to evaluate their efficacy on the retention ability of metal crowns to implant abutments.

## Materials and methods

The current research was conducted in the Prosthodontics Department, Inderprastha Dental College & Hospital, Ghaziabad, from April 2020 to February 2021.

**Figure 1:**
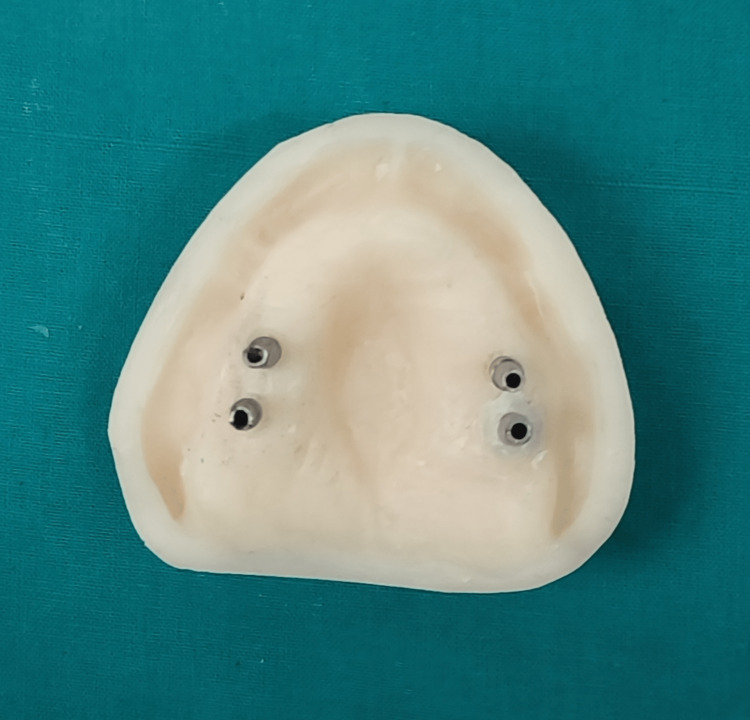
Implant analogs secured in epoxy resin casts

Four edentulous maxillary casts made of epoxy resin were created for this in vitro study. A mold was created using an acrylic resin model of an edentulous maxilla to create an epoxy resin cast. As per the manufacturer's instructions, a 10:1 mixture of the hardener (Arduur HY 951, Huntsman Advanced Materials LLC, Bengaluru, India) and epoxy resin (Araldite CY 230-1 IN, India) was used. The mixture was added to the mold and allowed to set. After 24 hours, the epoxy resin cast was created, finished, and polished. With the aid of a milling machine, holes for implant analogs were made in each of the four epoxy resin casts in the right and left first and second molar regions. Implant analogs (Internal Hex, Adin Dental Implant Systems Ltd., Tel-Aviv, Israel) were then secured with the aid of clear autopolymerizing acrylic resin (Rapid Repair, Pyrax Polymars, Roorkee, India) (Figure 1).

**Figure 2:**
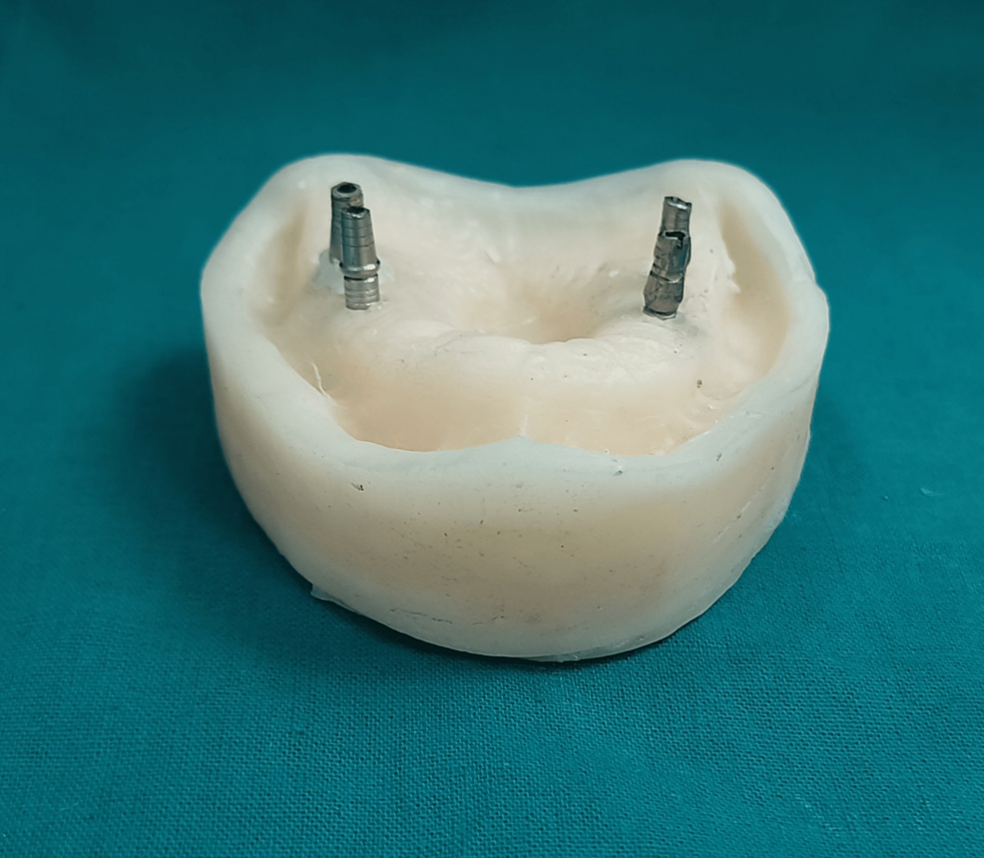
Implant abutments were screwed into each of the analogs in epoxy resin casts

With the aid of an Allen key (Adin Dental Implant Systems Ltd., Afula, Israel) and a torque wrench, the implant abutments, which have a diameter of 4 mm and a height of 11 mm, were screwed into each of the analogs (Figure 2).

Four metal crowns were then created for each epoxy resin cast (two on the left implant analog and two on the right implant analog in the first and second molar regions).

**Figure 3:**
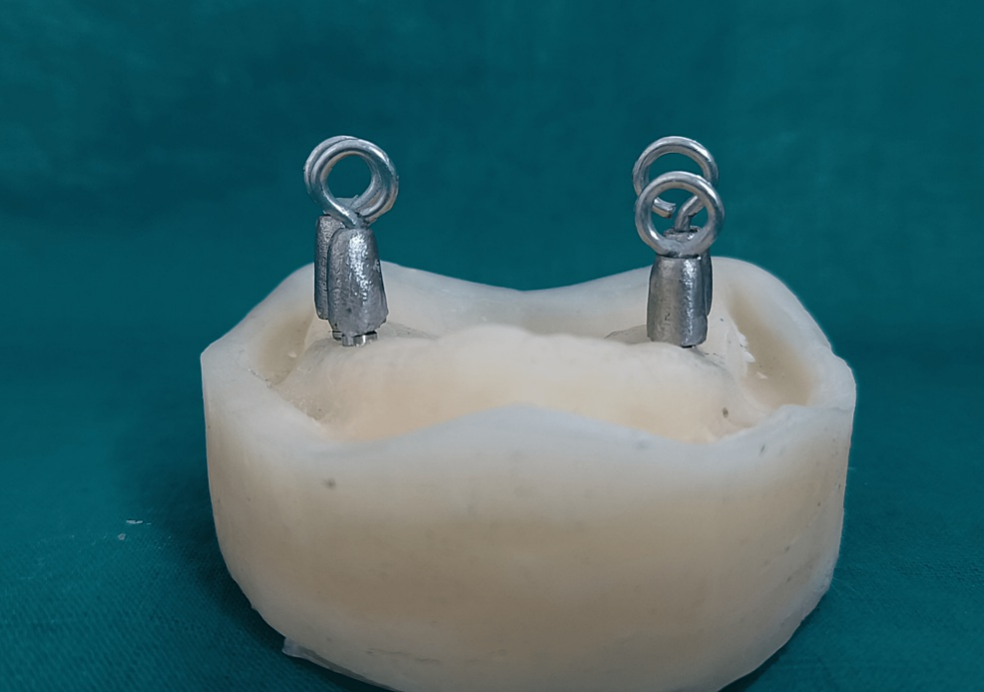
Castings were cemented using each luting cement in epoxy resin casts

The access holes in the abutments were sealed with clear autopolymerizing acrylic resin (Rapid Repair, Pyrax Polymars) after being filled with a cotton plug. Inlay wax was used to create a wax pattern of a crown after the die spacer and die lubricant had been applied (Bego, Bremen, Germany). Each crown's occlusal surface had a loop of wax added to it that was 8 mm in diameter for a retention test. The non-precious metal alloy (Wiron® 99, Bego) was cast using an induction casting machine after the wax patterns were invested in a phosphate-bonded investment (Bellavest®, Bego). The castings were polished and cemented using each luting cement (Figure 3).

Luting cements

**Figure 4:**
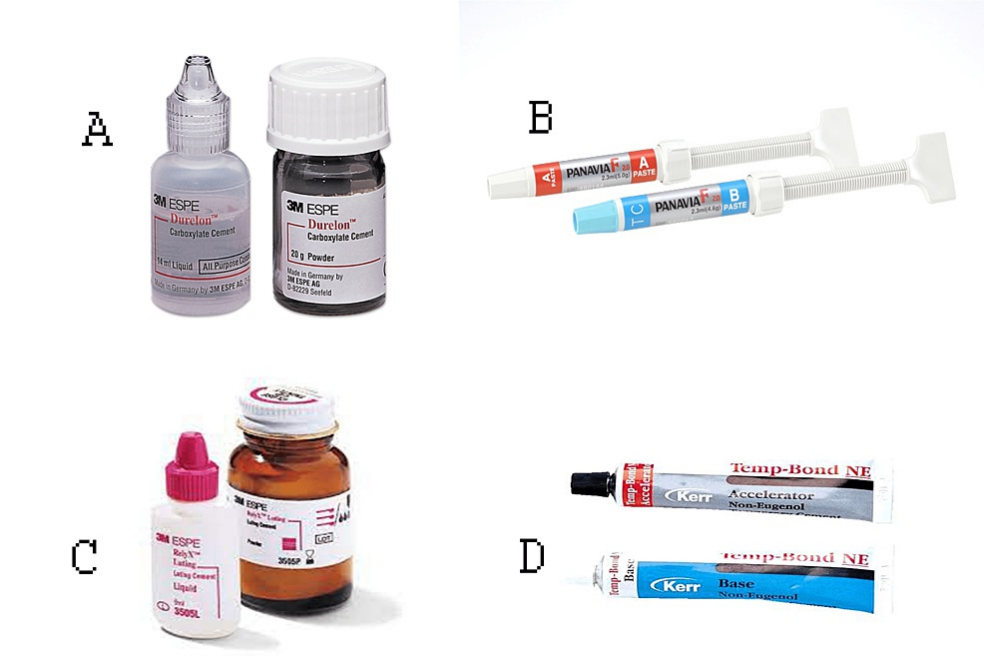
Luting cements Group A: polycarboxylate cement (DUR) (DurelonTM, 3M Espe, St. Paul, MN); Group B: PANAVIA™ F 2.0 dual-cure resin cement (Kuraray America, Inc., New York, NY); Group C: resin-modified glass ionomer (3M™ RelyX™ Luting, 3M Espe); and Group D: non-eugenol temporary resin cement (Kerr-Temp, KaVo Kerr, Brea, CA)

Group A: polycarboxylate cement (DUR) (DurelonTM, 3M Espe, St. Paul, MN); Group B: PANAVIA™ F 2.0 dual-cure resin cement (Kuraray America, Inc., New York, NY); Group C: resin-modified glass ionomer (3M™ RelyX™ Luting, 3M Espe); and Group D: non-eugenol temporary resin cement (Kerr-Temp, KaVo Kerr, Brea, CA) (Figure 4).

Excess cement had been scraped off with a curette. Twenty-four hours after cementation, the specimens were exposed to thermocycling with 10,000 cycles at +5º and +55º C, and then a cyclic compressive load of 100 N with a frequency of 0.72 Hz was applied to simulate two to three months of chewing (oral condition). Later, all the specimens were placed in artificial saliva (Moi Stir, Kingswood Laboratories, Inc., Indianapolis, IN) for 48 hours at 37 ºC. Using a universal testing machine, the dislodging forces of the copings were assessed after the aging process.

The highest tensile force (Mpa) required to debond each crown was taken into consideration when pulling off cemented crowns at a crosshead speed of 1 mm/min using Instron universal testing machine (TSI‑Tecsol, Bengaluru, India). Statistical analysis for obtained data was done using one-way ANOVA and the post-hoc Tukey test.

## Results

The average retentive strength in the groups was as follows - Group A: 531.362 ±24.642 Mpa; Group B: 674.065 ±18.432; Group C: 342.063 ±28.078; Group D: 138.256 ±3.561. Our findings showed that non-eugenol temporary resin implant cement has the lowest retention rate, followed by polycarboxylate luting cement, resin-modified glass ionomer cement, and resin cement (Table 1).

**Table 1:** Retentive strength for various luting cement groups The test used: ANOVA SD: standard deviation

Cement groups		Retentive strength, Mpa, mean ±SD	P-value
Group A	Polycarboxylate cement	531.362 ±24.642	0.001
Group B	Panavia F 2.0 dual cure resin cement	674.065 ±18.432	
Group C	Resin-modified glass ionomer	342.063 ±28.078	
Group D	Non-eugenol temporary resin cement	138.256 ±3.561	

One-way ANOVA was used to evaluate the average retention strength among the four groups, and the results showed that the F value was 1261.632 and the p-value was 0.001, which was extremely significant. The average variation among the retentive strengths of all four groups was greatly significant with a p-value of 0.001 when each group was compared with the other using post-hoc Tukey HSD (Table 2).

**Table 2:** Intergroup comparison of retentive strength for various luting cements *Highly significant The test used: post-hoc Tukey HSD GIC: glass-ionomer cement; HSD: Honest Significant Difference

Group comparison	Mean variation	P-value
Polycarboxylate vs. dual cure resin, Group A vs. Group B	142.703	<0.001*
Polycarboxylate vs. resin-modified GIC, Group A vs. Group C	189.299	<0.001*
Polycarboxylate vs. non-eugenol temporary, Group A vs. Group D	393.106	<0.001*
Dual cure resin vs. resin-modified GIC, Group B vs. Group Group C	332.002	<0.001*
Dual cure resin vs. non-eugenol temporary, Group B vs. Group D	535.809	<0.001*
Resin-modified GIC vs. non-eugenol temporary, Group C vs. Group D	203.807	<0.001*

## Discussion

Since dual cure resin cement (Panavia) is self-adhesive, it may have the highest retention strength in the current study. It adheres to the metal surface and forms a mechanical bond [3]. Our findings are consistent with those of Kapoor et al. and Pan et al., who concluded that resin cement had the greater retention value and non-eugenol acrylic-based temporary implant cement had the lowest retention value [3,5].

Using various luting agents, Nejatidanesh et al. assessed the retention values of metal copings supported by implants. They reported that the definitive cementation of single implant-supported restorations should be done using zinc phosphate, zinc polycarboxylate, resin cement, and resin-modified glass ionomer cements [6]. Ahsan et al. found that zinc phosphate cement had the next highest mean cement failure load, followed by resin‑bonded ZOE cement [7]. The retentiveness of specially formulated implant cements was tested by Sarfaraz et al. using non-eugenol zinc oxide luting cement. They concluded that non-eugenol temporary resin cement had the greatest tensile strength, followed by non-eugenol zinc oxide cement, while resin-based acrylic urethane cement had the least tensile strength [2].

According to Sheets et al.'s analysis, there was no discernible variation between non-eugenol zinc oxide cement and non-eugenol temporary resin cement [8]. ZOE cement has been shown to have high solubility in direct contact with water and needs enough time for a full setting reaction [9]. Glass ionomer can be used as semi-permanent cement for both single crowns (SCs) and fixed dental prostheses (FDPs), as per Bishti et al.'s analysis of three different cements for implant-supported SC retention forces [1]. According to some reports, the proportion of retention loss following compressive cyclic loading is lower if the initial cement retention is higher [4,10,11,12]. For the purpose of cementing implant crowns, Garg et al. compared resin-bonded ZOE cement, zinc phosphate cement, glass ionomer cement, and zinc polycarboxylate cement, and came to the conclusion that these cements were superior over others for cementation [13]. Zinc polycarboxylate cement, glass ionomer, and zinc phosphate were found to have the highest retentive values by Sathyanarayan et al. [14].

Retention force and cement type, taper, internal surface cleaning, abutment surface, crown pretreatment, and cement gap have all been shown to significantly correlate with one another [15]. Deepthi et al. found that non-eugenol zinc oxide cement had the least retention compared to zinc phosphate cement and glass ionomer cement and stated that non-eugenol zinc oxide cement may not be suitable for luting single-unit implant-supported restorations [15]. Özyılmaz et al. assessed the retention strength of various types of cements - Poly F (PF), GC FujiCEM (GCF), Rely X (RX) - and concluded that there was no considerable variation observed between RX and MCS and retention improved with increasing the cement gap from 20 to 40 µm [16]. Ahmed et al. assessed the retention values and marginal adaptation of implant-supported metal copings using different luting agents. They concluded that resin cement, resin-modified glass ionomer, and zinc phosphate had statistically the same retentive quality [17]. Resin cement has a retention strength almost twice that of resin-modified glass ionomer [18].

In the present study, the highest tensile strength was found with polycarboxylate and glass ionomer cement since they have chemical adhesion properties, while dual-cure resin had the highest retentive and self-adhesion capacity. Non-eugenol temporary resin cement is designed for implant-retained crowns and provisionals since it provides secure retention and excellent marginal seal. It is a very tough resin, which uses mechanical retention to adhere the crown to the abutment. Yet, the restoration can be removed easily when desired due to its unique elasticity. However, it has the lowest retentive strength [3].

According to the manufacturer, resin cement can be used as semi-permanent cement for customized abutments and has a lower dislocation resistance than conventional cement. Variability in the data across studies may be attributed to variations in experimental conditions, cement formulation/composition, abutment characteristics, and prosthesis design. The present study found that non-eugenol temporary resin implant luting cement had the lowest retention value among the tested cements and the restoration can be removed easily due to its unique elasticity; hence it helps in easy retrievability of the prosthesis in cases of future failure. However, several other studies have stated that resin cements are useful for the cementation of single-implant prostheses for better retention [16]. The clinician should exercise great caution in the choice of cement as there is a high risk of component loosening based on the findings of the current study.

This study has a few limitations, primarily the absence of a humidor and the inability to perform thermocycling; we also failed to account for the potential effects of degradation that might occur over time in a clinical setting. Further research is required to confirm our findings.

## Conclusions

The selection of cement is crucial for achieving an appropriate level of retention of the implant prosthesis while allowing for removal, thereby increasing the longevity of implant prostheses. Our findings showed that non-eugenol temporary resin implant luting cement has the lowest retention value among all groups, followed by resin-modified glass ionomer cement, polycarboxylate luting cement, and resin cement. Non-eugenol temporary resin implant cement enables simple retrievability of the prosthesis in the event of a future failure and is appropriate for implant restorations with cement retention. Based on our findings, cements made of polycarboxylate and resin have the highest retention strength.
